# Pregnancy and acromegaly

**DOI:** 10.1007/s11102-016-0740-3

**Published:** 2016-08-27

**Authors:** Ammar Muhammad, Sebastian J. Neggers, Aart J. van der Lely

**Affiliations:** 1 Rotterdam Pituitary Centre, Erasmus University MC, Rotterdam, The Netherlands; 2 Section of Endocrinology, Department of Medicine, Erasmus University MC, P.O. Box 2040, 3000 AC Rotterdam, The Netherlands

**Keywords:** Acromegaly, Pregnancy, Review, Treatment, Complications

## Abstract

**Introduction:**

Acromegaly is a rare disorder in which, due to the high incidence of secondary hypogonadism, pregnancies are relatively rare. However, some women with acromegaly do get pregnant, which brings along questions about medication, complications and follow-up. This review tries to address these issues and provide the reader with practical information.

**Methods:**

This review summarizes published data.

**Conclusions:**

Acromegaly is a disorder that is characterized by changes in growth hormone (GH), insulin-like growth factor-1 (IGF-1) and insulin concentrations and actions. All these hormones are important in pregnancy as well. In principle, the fetal-placental collaboration between mother and child more-or-less takes over the control over GH and IGF-1, not only in normal physiology but also to a certain extend in acromegaly. When medication for the high GH levels or actions is continued during pregnancy, both dopamine agonists, somatostatin analogs and GH receptor antagonists have been used and the available data suggest that there are no adverse consequences on mother or fetus to date. However, it is strongly advised to stop any medical intervention during pregnancy until more data are available on the safety of these compounds. Also, medical treatment is not needed as tumor size and disease activity are not reported to escape.

## Introduction

Acromegaly is a rare syndrome that results when the anterior pituitary gland produces excess growth hormone (GH) after epiphyseal plate closure at puberty. The disorder is further characterized by changes in insulin-like growth factor-1 (IGF-1) and insulin concentrations and actions. During pregnancy, these hormones are important in pregnancy as well. Also other processes, as methylation play an important role in the fetal-placental interplay [1].

In normal pregnancy, the physiological increase in estrogen and progesterone decrease the sensitivity of the liver [2]. During the second half of normal pregnancy, the human placenta secretes placental GH in increasing amounts up to delivery while at the same time, pituitary GH secretion is progressively suppressed [3]. These two changes are probably the main reasons behind the apparent lack of biochemical escape in pregnant acromegaly patients when medical treatment is stopped [4].

Therefore, it is commonly not necessary to continue medical treatment for active acromegaly during pregnancies. In this review, we try to address the (patho)physiological metabolic background of normal pregnancy versus pregnancy in acromegaly patients. Also we discuss the available safety data of medical treatment modalities for acromegaly.

## What is different and what is identical in the hormonal patterns between normal pregnancies and the ones in acromegaly

In a nice overview by Verhaeghe [5], it is explained in detail that normal pregnancies are accompanied by notable changes in the secretion of GH and IGFs. Placental GH (pGH) is discernible in maternal plasma from early pregnancy, rising exponentially until 37 weeks. Meanwhile, pituitary GH gradually drops to near-undetectable levels. While there might be a modest reduction in circulating IGF-I in early pregnancy, IGF-I increases two- to three-fold in the second half, again with a peak at around 37 weeks. Thus, placental GH is believed to replace pituitary GH as the primary stimulus for IGF-I secretion in pregnancy [5]. IGF-II concentrations also appear to show a modest (20–25 %) increase in the course of pregnancy [5]. All-in-all, during normal pregnancy a ‘gestational acromegaly’ develops in order to foster fetoplacental growth.

Placental and maternal pituitary hormones tightly regulate fetal growth and placental GH mobilizes maternal nutrients for fetal growth by inducing maternal insulin resistance [6]. However, the adenomatous tumor cells appear to be resistant to the factors that usually inhibit pituitary GH secretion during the second part of normal pregnancy. In acromegaly, the decrease of IGF-I in the first trimester of pregnancy may be related to decreased production or increased turnover rather than to a marked decrease in pituitary GH secretion [7]. Also, high estradiol levels interfere with the hepatic production of IGF-I during the first part of pregnancy. This effect, however, seems to be overridden by the progressive secretion of placental GH [7]. This predominant effect of placental GH on maternal IGF-I in late pregnancy is also nicely illustrated by the marked drop of IGF-I 2 days after delivery [7].

In normal pregnancy, the physiological increase in estrogen and progesterone has known consequences for the sensitivity of the liver for GH, as was recently described and confirmed by Persechini et al. [2]. While this phenomenon is present in normal pregnancies, it’s also present in acromegaly. Administration of high doses of estrogens to patients with acromegaly has been shown already more than 50 years ago to improve symptomatology of acromegaly and glucose tolerance [8]. Selective estrogen receptor modulators (SERMs) mimic the effects of estrogen in bone, liver and the cardiovascular system, but act as anti-estrogens in endometrial and breast tissue [9]. Balili and Barkan [9] evaluated hormonal effects of the SERM tamoxifen in 15 male acromegaly patients with active disease and 2 post-menopausal women, also with biochemically-active acromegaly despite the fact that other modalities were ineffective in normalizing their IGF-1 levels. They observed that tamoxifen did not affect basal GH secretion, but it decreased circulating IGF-I in 14 patients (82 %) and normalized plasma IGF-I in 8 patients (47 %) [9].

During the second half of normal pregnancy, the human placenta secretes its specific GH variant (placental GH; pGH) in increasing amounts up to delivery. During the same period, pituitary GH secretion is progressively suppressed [3]. Noteworthy is that the high homology of GH with pGH frequently results in falsely high GH levels in competitive immunoassays during pregnancy [10]. However, in immunometric assays, falsely high or low GH levels can result from GH-like molecules binding to both or only one monoclonal antibody [10]. Dias et al. [10] evaluated potential negative interference of pregnancy serum in their GH-IFMA assay. Addition of pregnancy serum to ‘acromegaly’ serum resulted in a marked decrease in GH, but addition of pGH did not change GH measurements [10]. Therefore, GH measurements in pregnancy by immunometric assays must be made after exclusion of pregnancy serum interference by dilutional tests [10].

Ringholm and coworkers evaluated whether levels of pGH and IGF-I are associated with development of large for gestational age (LGA) infants in pregnant women with type-1 diabetes [11]. Women delivering LGA infants had lower placental GH levels in early pregnancy [11]. Apparently, growth factors and maternal weight gain in early pregnancy may be important for healthy fetal growth. Therefore, the lack of fetal abnormalities in children born out of acromegaly mothers shows that, apparently, the normal physiological drivers seem to control the autonomous GH secretion by the somatotropinoma.

The human placental lactogen (hPL) gene family consists of two GH and three PL genes [12]. pGH expressed by the placenta, becomes the predominant GH in the mother. hPL, which is the product of the hPL-A and hPL-B genes, is secreted into both the maternal and fetal circulations after the sixth week of pregnancy [12]. pGH and hPL act in concert in the mother to stimulate IGF-I and modulate intermediary metabolism, resulting in an increase in the availability of glucose and amino acids to the fetus. hPL acts via lactogenic receptors and possibly a unique PL receptor in the fetus. This modulates embryonic development, regulate intermediary metabolism and stimulate the production of IGFs, but also pulmonary surfactant [12].

**Fig. 1:**
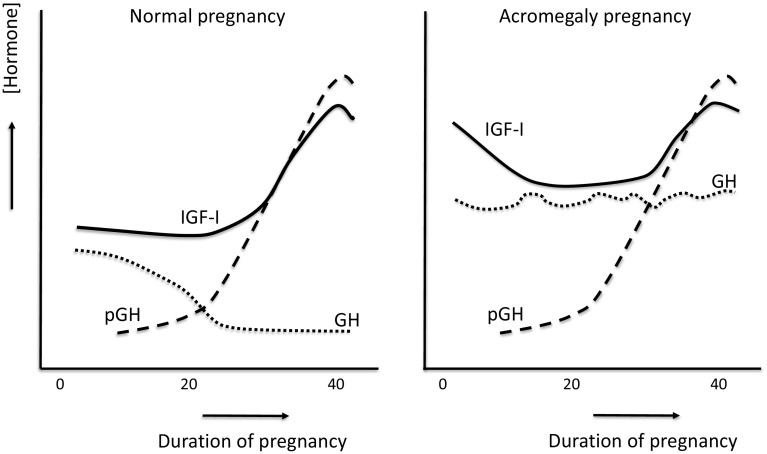
Semi-qualitative conceptual changes in GH, IGF-I and placental GH (pGH) during normal pregnancy and during pregnancy in women with acromegaly, based on [4, 5, 7]

The conceptual changes during pregnancy in normal subjects and women with acromegaly are shown in Fig. 1.

## What happens if medical treatment is stopped?

The reasons behind the apparent lack of biochemical escape in pregnant acromegaly patients when medical treatment is stopped were already addressed by Beckers et al. [4]. They described 2 young women who remained acromegalic despite transsphenoidal removal of their pituitary adenomas. Increased basal levels of GH and IGF-1 as well as paradoxical GH release after TRH injection were noted [4]. Both women became pregnant and delivered term babies without any complication. In both patients, pituitary GH remained elevated during the entire pregnancy, contrary to the situation in normal women. Tissue placental GH concentrations were within the range of levels in normal placentas. An increase in serum IGF-1 in late pregnancy was also similar to that observed in normal pregnancy. This confirms that increased IGF-I levels are not pituitary GH dependent in late pregnancy. Apparently, adenomatous somatotrophs lack an IGF-I-dependent feedback regulation present in normal somatotrophs [4].

Most acromegaly patients who become pregnant while on medical therapy in fact represent patients in which the fetus encounters the compound that is used by the mother for at least the first 6 weeks or so. To our knowledge no controlled data exist of women who preconceptionally stopped medication to prevent exposure of the fetus. Also there is a complete lack of data on safety in those who start taking medication to control acromegaly, as the indication for this is extremely rare.

Because of the overlap between the normal gestational acromegaly and pregnancy in acromegaly, the distinction between the two more or less disappears. In a recent prospective study in 10 pregnancies in 8 acromegaly patients, in whom medication for acromegaly was stopped immediately following confirmation of pregnancy, Dias et al. [13] describe that no tumor growth was observed. Nine deliveries were at term and one at 35 weeks (preeclampsia). No fetal abnormalities were observed. Mean IGF-1 levels before and during pregnancy were similar, but increased significantly during puerperium [13]. As IGF-1 in normal pregnancies is already increased after midgestation, the prevalence of controlled IGF-1 in those 10 pregnancies rose significantly from 2/10 (<20 weeks) to 9/10 (>30 weeks) [13].

But even in the case that GH and IGF-1 don’t become normal during pregnancy in at least a subsection of subjects, the fetus is protected for this as GH and IGF-1 don’t seem to cross the placenta [14].

Tumor size is another important issue in acromegaly. Stopping medication might have clinical consequences for the patient when tumor size increases. Only very few patients with tumors that secrete GH have been reported to have had enlargement of their tumors during pregnancy [15, 16]. Therefore, in a review by Molitch, he concludes that surgery or medical therapy for somatotropinomas and for clinically nonfunctioning adenomas is almost never indicated during pregnancy [17].

In a recent review Cheng et al. [18] also reported that most patients with acromegaly during pregnancy do not have an increase in tumor size, and metabolic complications are uncommon.

Caron et al. [19] reported on a retrospective multicenter study of 59 pregnancies in 46 women with acromegaly that pregnancy in women with active or uncontrolled acromegaly may be associated with an increased risk of as already mentioned not only gestational diabetes but also gravid hypertension. The IGF-axis may be also implicated in glucose homeostasis, but its longitudinal profile across gestation in relation to the development of gestational diabetes (GDM) is largely unknown [20]. Zhu et al. prospectively investigated IGF-axis biomarkers in early-to-mid pregnancy in relation to subsequent GDM risk in a case–control study of 107 GDM cases and 214 non-GDM controls. They showed that the IGF-axis, IGFBP-2 in particular, may be implicated in the pathogenesis of GDM with significant associations and incremental predictive value detected as early as the end of the first trimester, approximately 10–18 weeks earlier before GDM is typically screened for. The risk of GDM seems to be increased in acromegaly as GDM was diagnosed in four of the 59 pregnancies (6.8 %) in the series reported by Caron et al. [19]. Three of these four women did not have diabetes mellitus before pregnancy, whereas acromegaly was diagnosed during pregnancy in the fourth woman, and the existence of diabetes mellitus before pregnancy could not be ascertained [19].

Cheng et al. [21] report in 13 newly described pregnancies along with systematic review of an additional 34 cases that pregnancy in treated acromegalic women can proceed without significant complications or teratogenicity.

## Safety aspects of using dopamine agonists during pregnancy

Cheng et al. [18] report to have found that dopamine agonists, somatostatin analogues, and pegvisomant have been reported to be safe during pregnancy.

Most data on safety of the use of dopamine agonists during 61 pregnancies in 50 women treated with cabergoline were obtained by Ricci et al. from women with prolactinomas, however [22]. Short-term treatment with bromocriptine has not been found to cause any increase in spontaneous abortions, ectopic pregnancies, trophoblastic disease, multiple pregnancies, or congenital malformations [23–25]. Yap et al. [26] reported a case of acromegaly diagnosed in the second trimester of pregnancy, in which bromocriptine corrected visual field defects and suppressed prolactin secretion but did not reduce fasting growth hormone levels. In 1976 already, Aono et al. [27] reported a case of an acromegalic patient with galactorrhea-amenorrhea who conceived following long-term bromocriptine therapy. They showed that bromocriptine can restore ovulatory function not only to the patient with a hypothalamic disorder but also to acromegalic patients.

Experience with the use of cabergoline in pregnancy is limited [28]. Ricci et al. [28] collected information on 61 pregnancies in 50 women treated with cabergoline. These pregnancies resulted in 12 (19.7 %) early terminations (five induced abortions, six spontaneous abortions, one hydatidiform mole) and 49 (80.3 %) live births. Their data in combination with previous reports can more or less exclude a major congenital malformation risk >10 % associated with pregnancy exposure to cabergoline [28–30].

## Safety aspects of using somatostatin analogs during pregnancy

Maffei et al. [31] reported a case study on the effects of octreotide, a somatostatin receptor ligand (SRL) on uterine artery blood flow, which they addressed as acute, reversible, and clinically irrelevant hemodynamic changes in the maternal-fetal barrier. A certain degree of mother-to-fetus transplacental passage of at least octreotide seems to occur by passive diffusion [31, 32]. Fassnacht et al. reported a 24-year-old woman with active acromegaly despite pituitary surgery and irradiation who received continuous octreotide LAR treatment for the control of GH excess throughout her pregnancy. The patient delivered a healthy girl following an uneventful pregnancy [33]. Takano et al. reported a 35-year-old woman with active acromegaly who also received continuous octreotide LAR treatment for the control of GH excess until discovery of her pregnancy. The patient delivered a healthy boy following an uneventful pregnancy after discontinuing octreotide LAR as soon as possible at the early phase of pregnancy [34].

Neal [35] reported a successful pregnancy in a 43-year-old woman with acromegaly and treatment during pregnancy with octreotide. This patient decided to continue octreotide therapy because of the relatively large size of the tumor and severe headaches when use of the medication was discontinued. Interestingly, GH and IGF-I levels remained normal throughout pregnancy, and a normal full-term infant was born. In their review they also reported about 6 other women, who used octreotide during the first trimester of pregnancy in seven other women with acromegaly (and throughout pregnancy in one) [35]. No adverse effects on the fetus were observed [35]. Also, lanreotide was also used in one patient briefly during the first trimester without adverse consequences [35].

Caron et al. reported four women who gave birth to a small-for-gestational-age infant without gross malformations while receiving a somatostatin analog alone (n = 2) or combined with a dopamine agonist (n = 2) during <2 months of pregnancy [11]. Short-acting octreotide induce an acute, reversible, and clinically irrelevant hemodynamic changes in the uterine artery [31]. Whether or not these effects of octreotide are responsible for the microsomia that Caron et al. observed is unclear.

## Safety aspects of using pegvisomant during pregnancy

Van der Lely et al. [36] summarized all available data on pregnancy outcome of 35 casus of acromegaly patients exposed to pegvisomant (PEGV) during pregnancy as present in the Pfizer’s Global Safety Database up to March 2014.

Although the number of reported pregnancies with exposure to PEGV was very small, the presented data reflect the largest series of data available to date and do not suggest adverse consequences of PEGV on pregnancy outcome [36]. Nevertheless, they stressed out that PEGV should not be used during pregnancy unless absolutely necessary. Riddle Brian et al. already reported a case of a 26-year-old female with acromegaly who had failed surgical and subsequent medical therapy but whose disease was well controlled on PEGV [37]. The patient conceived and was continued on PEGV throughout pregnancy. They also collected both maternal and cord blood samples at parturition, and later analyzed her breast milk.

They observed that maternal IGF-I was well controlled during gestation and fetal GH and IGF-1 were within the normal range. Maternal PEGV levels were consistent with a 25 mg daily dosage. Fetal- and breast milk PEGV levels appeared to be minimal and near the range that can be detected in untreated acromegalic patients [37].

## Conclusions

Acromegaly is characterized by changes in concentrations and actions of GH, IGF-1 and insulin. All these hormones play an essential role in pregnancy as well. In principle, the placenta takes over the control over maternal GH and IGF-1 secretion, not only in normal physiology but also to a certain extend in acromegaly.

During normal pregnancy, placental GH rises exponentially until 37 weeks. Meanwhile, pituitary GH gradually drops to near-undetectable levels. While there might be a modest reduction in circulating IGF-I in early pregnancy, IGF-I increases two- to three-fold in the second half, again with a peak at around 37 weeks.

The question is whether or not patients with acromegaly should be treated for the disease during pregnancy anyway. The consensus is, however, that there is no indication to use medication to control GH hypersecretion or tumor size in acromegaly patients during pregnancy.

When for whatever reason medication for the high GH levels or actions is continued during pregnancy, both dopamine agonists, somatostatin analogs and growth hormone receptor antagonists have been used and the available data suggest that there are no adverse consequences on mother or fetus to date, although again it is generally advised to stop any medical intervention during pregnancy as stopping until postpartum reactivation of the disease activity has been proven to be safe for mother and child. Also, more data on the safety of these compounds are lacking.

This results in the following recommendations:

- When practical feasible, women could stop medication before conception, although data to support a positive effect of this are lacking. This also accounts for male acromegaly fathers to be.
- When pregnancy is confirmed, medical treatment can be stopped in almost every patient, as biochemical escape is very unlikely, which also accounts for tumor size.
- When need for medical intervention is indicated to control e.g. tumor-size or severe headaches, all available medical treatment modalities seem to be safe enough, although large series are lacking.
- In order to detect increases in tumor-size early enough, visual field assessments are indicated every trimester, but only in those subjects with large tumors that extend to the optic chiasm only. When visual fields become abnormal, this must be followed by an MRI of the sellar region.
- When (although very uncommon) an unacceptable increase tumor size is detected, transsphenoidal surgery might be indicated.

## References

[1] Nawathe AR, Christian M, Kim SH, Johnson M, Savvidou MD, Terzidou V (2016). Insulin-like growth factor axis in pregnancies affected by fetal growth disorders. Clin Epigenetics.

[2] Persechini ML, Gennero I, Grunenwald S, Vezzosi D, Bennet A, Caron P (2015). Decreased IGF-1 concentration during the first trimester of pregnancy in women with normal somatotroph function. Pituitary.

[3] Scippo ML, Frankenne F, Hooghe-Peters EL, Igout A, Velkeniers B, Hennen G (1993). Syncytiotrophoblastic localization of the human growth hormone variant mRNA in the placenta. Mol Cell Endocrinol.

[4] Beckers A, Stevenaert A, Foidart JM, Hennen G, Frankenne F (1990). Placental and pituitary growth hormone secretion during pregnancy in acromegalic women. J Clin Endocrinol Metab.

[5] Verhaeghe J (2008). Does the physiological acromegaly of pregnancy benefit the fetus?. Gynecol Obstet Investig.

[6] Newbern D, Freemark M (2011). Placental hormones and the control of maternal metabolism and fetal growth. Curr Opin Endocrinol Diabetes Obes.

[7] Wiesli P, Zwimpfer C, Zapf J, Schmid C (2006). Pregnancy-induced changes in insulin-like growth factor I (IGF-I), insulin-like growth factor binding protein 3 (IGFBP-3), and acid-labile subunit (ALS) in patients with growth hormone (GH) deficiency and excess. Acta Obstet Gynecol Scand.

[8] Clemmons DR, Underwood LE, Ridgway EC, Kliman B, Kjellberg RN, Van Wyk JJ (1980). Estradiol treatment of acromegaly. Reduction of immunoreactive somatomedin-C and improvement in metabolic status. Am J Med.

[9] Balili I, Barkan A (2014). Tamoxifen as a therapeutic agent in acromegaly. Pituitary.

[10] Dias ML, Vieira JG, Abucham J (2013). Detecting and solving the interference of pregnancy serum, in a GH immunometric assay. Growth Horm IGF Res.

[11] Ringholm L, Juul A, Pedersen-Bjergaard U, Thorsteinsson B, Damm P, Mathiesen ER (2015). Lower levels of placental growth hormone in early pregnancy in women with type 1 diabetes and large for gestational age infants. Growth Horm IGF Res.

[12] Freemark M (2006). Regulation of maternal metabolism by pituitary and placental hormones: roles in fetal development and metabolic programming. Horm Res.

[13] Dias M, Boguszewski C, Gadelha M, Kasuki L, Musolino N, Vieira JG, Abucham J (2014). Acromegaly and pregnancy: a prospective study. Eur J Endocrinol.

[14] Frankenne F, Closset J, Gomez F, Scippo ML, Smal J, Hennen G (1988). The physiology of growth hormones (GHs) in pregnant women and partial characterization of the placental GH variant. J Clin Endocrinol Metab.

[15] Kupersmith MJ, Rosenberg C, Kleinberg D (1994). Visual loss in pregnant women with pituitary adenomas. Ann Intern Med.

[16] Okada Y, Morimoto I, Ejima K, Yoshida K, Kashimura M, Fujihira T, Eto S (1997). A case of active acromegalic woman with a marked increase in serum insulin-like growth factor-1 levels after delivery. Endocr J.

[17] Molitch ME (2003). Pituitary tumors and pregnancy. Growth Horm IGF Res.

[18] Cheng V, Faiman C, Kennedy L, Khoury F, Hatipoglu B, Weil R, Hamrahian A (2012). Pregnancy and acromegaly: a review. Pituitary.

[19] Caron P, Broussaud S, Bertherat J, Borson-Chazot F, Brue T, Cortet-Rudelli C, Chanson P (2010). Acromegaly and pregnancy: a retrospective multicenter study of 59 pregnancies in 46 women. J Clin Endocrinol Metab.

[20] Zhu Y, Mendola P, Albert PS, Bao W, Hinkle SN, Tsai MY, Zhang C (2016). Insulin-like growth factor axis and gestational diabetes: a longitudinal study in a multiracial cohort. Diabetes.

[21] Cheng S, Grasso L, Martinez-Orozco JA, Al-Agha R, Pivonello R, Colao A, Ezzat S (2012). Pregnancy in acromegaly: experience from two referral centers and systematic review of the literature. Clin Endocrinol.

[22] Molitch ME (2015). Endocrinology in pregnancy: management of the pregnant patient with a prolactinoma. Eur J Endocrinol.

[23] Krupp P, Monka C (1987). Bromocriptine in pregnancy: safety aspects. Klin Wochenschr.

[24] Krupp P, Ruch R, Turkalj I (1985). Drugs in pregnancy: assessment of Parlodel. Prog Clin Biol Res.

[25] Turkalj I, Braun P, Krupp P (1982). Surveillance of bromocriptine in pregnancy. JAMA.

[26] Yap AS, Clouston WM, Mortimer RH, Drake RF (1990). Acromegaly first diagnosed in pregnancy: the role of bromocriptine therapy. Am J Obstet Gynecol.

[27] Aono T, Shioji T, Kohno M, Ueda G, Kurachi K (1976). Pregnancy following 2-bromo-alpha-ergocryptine (CB-154)-induced ovulation in an acromegalic patient with galactorrhea and amenorrhea. Fertil Steril.

[28] Ricci E, Parazzini F, Motta T, Ferrari CI, Colao A, Clavenna A, Rocchi F, Gangi E, Paracchi S, Gasperi M, Lavezzari M, Nicolosi AE, Ferrero S, Landi ML, Beck-Peccoz P, Bonati M (2002). Pregnancy outcome after cabergoline treatment in early weeks of gestation. Reprod Toxicol.

[29] Auriemma RS, Perone Y, Di Sarno A, Grasso LF, Guerra E, Gasperi M, Pivonello R, Colao A (2013). Results of a single-center observational 10-year survey study on recurrence of hyperprolactinemia after pregnancy and lactation. J Clin Endocrinol Metab.

[30] Domingue ME, Devuyst F, Alexopoulou O, Corvilain B, Maiter D (2014). Outcome of prolactinoma after pregnancy and lactation: a study on 73 patients. Clin Endocrinol.

[31] Maffei P, Tamagno G, Nardelli GB, Videau C, Menegazzo C, Milan G, Calcagno A, Martini C, Vettor R, Epelbaum J, Sicolo N (2010). Effects of octreotide exposure during pregnancy in acromegaly. Clin Endocrinol.

[32] Caron P, Gerbeau C, Pradayrol L (1995). Maternal-fetal transfer of octreotide [letter; comment]. N Engl J Med.

[33] Fassnacht M, Capeller B, Arlt W, Steck T, Allolio B (2001). Octreotide LAR treatment throughout pregnancy in an acromegalic woman. Clin Endocrinol.

[34] Takano T, Saito J, Soyama A, Ito H, Iizuka T, Yoshida T, Nishikawa T (2006). Normal delivery following an uneventful pregnancy in a Japanese acromegalic patient after discontinuation of octreotide long acting release formulation at an early phase of pregnancy. Endocr J.

[35] Neal JM (2000). Successful pregnancy in a woman with acromegaly treated with octreotide. Endocr Pract.

[36] van der Lely AJ, Gomez R, Heissler JF, Akerblad AC, Jonsson P, Camacho-Hubner C, Koltowska-Haggstrom M (2015). Pregnancy in acromegaly patients treated with pegvisomant. Endocrine.

[37] Riddle Brian S, Bidlingmaier M, Wajnrajch MP, Weinzimer SA, Inzucchi SE (2007). Treatment of acromegaly with pegvisomant during pregnancy: maternal and fetal effects. J Clin Endocrinol Metab.

